# Evolution of the Order Urostylida (Protozoa, Ciliophora): New Hypotheses Based on Multi-Gene Information and Identification of Localized Incongruence

**DOI:** 10.1371/journal.pone.0017471

**Published:** 2011-03-08

**Authors:** Zhenzhen Yi, Weibo Song

**Affiliations:** 1 Laboratory of Protozoology, Institute of Evolution & Marine Biodiversity, Ocean University of China, Qingdao, China; 2 Laboratory of Protozoology, Key Laboratory of Ecology and Environmental Science in Guangdong Higher Education, College of Life Science, South China Normal University, Guangzhou, China; Université Paris Sud, France

## Abstract

Previous systematic arrangement on the ciliate order Urostylida was mainly based on morphological data and only about 20% taxa were analyzed using molecular phylogenetic analyses. In the present investigation, 22 newly sequenced species for which alpha-tubulin, SSU rRNA genes or ITS1-5.8S-ITS2 region were sampled, refer to all families within the order. Following conclusions could be drawn: (1) the order Urostylida is not monophyletic, but a core group is always present; (2) among the family Urostylidae, six of 10 sequenced genera are rejected belonging to this family; (3) the genus *Epiclintes* is confirmed belonging to its own taxon; (4) the family Pseudokeronopsidae undoubtedly belongs to the core portion of urostylids; however, some or most of its members should be transferred to the family Urostylidae; (5) Bergeriellidae is confirmed to be a valid family; (6) the distinction of the taxon Acaudalia is not supported; (7) the morphology-based genus *Anteholosticha* is extremely polyphyletic; (8) ITS2 secondary structures of *Pseudoamphisiella* and *Psammomitra* are rather different from other urostylids; (9) partition addition bootstrap alteration (PABA) result shows that bootstrap values usually tend to increase as more gene partitions are included.

## Introduction

Ciliates, eurychoric unicellular eukaryotes, are characterized by complexes of cilia and a nuclear dimorphism [1]. In last 25 years, molecular phylogenetic analyses, especially based on small subunit rRNA (SSU rRNA) gene sequences, provided resolution of a number of important questions on the phylogenetic relationships within this group (for example, [2], [3]–[7]). However, many questions remain open, mostly related to a number of spirotrichean lineages, either on the assignment of certain species to one or another group or, more importantly, on the phylogenetic relationships within certain orders/families that contain a large number of taxa.

Among these, the order Urostylida is one of the most confused and diverse and is increasingly attractive for the researchers working on morphogenetic, taxonomic and molecular fields (for example, [4], [8], [9]–[13]). The most important apomorphy for urostylids is a zig-zagging ventral cirral pattern originating from more than six anlagen evolved possibly convergently for several times (for example, [ 1,9]). There are more than ten studies that include details of interrelationships within this order (for example, [ 1], [9], [14]–[21]), which are mainly based on morphological/morphogenetic data, but none reaches the same conclusions as another.

In his monograph of the Urostyloidea, Berger [9] recognised 154 valid species, and assigned most of them to four families (Holostichidae, Bakuellidae, Urostylidae and Epiclintidae) using the frontal ciliature and the midventral complex as the main features. Recently, another systematic classification was proposed by Lynn [1], which also divided the order Urostylida into four families (Epiclintidae, Pseudokeronopsidae, Pseudourostylidae, Urostylidae). Between these two systems, there is only agreement over the classification of Epiclintidae. In order to investigate further the evolutionary relationships among the urostylids, molecular phylogenetic analyses based on SSU rRNA gene sequences have been increasingly used in recent few years [4], [12], [22]–[27]. Although these investigations undoubtedly show that Urostylida is a large group within the Hypotricha, the monophyly of this order is not yet certain, and relationships within it are still confused. Furthermore, molecular phylogenies based on other gene markers, albeit with sparse taxon sampling, have produced rather different results compared to SSU rRNA phylogenies [22], [28], [29].

Comparison between different molecular trees is an essential step to reveal the evolution within investigated groups, even when independent datasets yield congruent results. The combined phylogenetic analyses of multiple genes have become popular due to the poor resolution of phylogenies based on single loci [30], and have successfully inferred better-resolved phylogenies within the major taxonomic groups, including animals [31], plants [32], fungi [33] and bacteria [34]. However, there are few ciliate phylogenies based on combined gene partitions [6]. With the advent of multi-gene phylogenies, particular emphasis has been placed on congruence or combinability of independent and possibly heterogeneous datasets [35]–[37]. To date, the only molecular urostylid phylogeny based on combined genes is that of Hewitt et al. [26] who used SSU-5.8S-LSU rRNA. There are only three congruent phylogenies, based on different genes that include few urostylid taxa [38]–[40].

The present study was initiated to improve our understanding of evolutionary relationships within the order Urostylida by extending the SSU rRNA gene, ITS1-5.8S-ITS2 region, and alpha-tubulin gene database. Moreover, molecular phylogenies are discussed with critical consideration of the taxonomic literature. In addition, statistical tests, i.e. incongruence length difference (ILD) test, Shimodaira-Hasegawa (S-H test) and partition addition bootstrap alteration (PABA) approach, are performed to detect incongruence among these three gene partitions.

## Results

### Analyses of Sequences and Secondary Structures

A total of one SSU rRNA gene, eight ITS1-5.8S-ITS2 regions, and 13 alpha-tubulin genes were sequenced in our analyses (Table 1).

**Table 1:** Urostylid Species for Which SSU rRNA Gene, ITS1-5.8S-ITS2 Regions and Alpha-Tubulin Gene Were Newly Sequenced in the Present Work.

Species	SSU rRNA gene		ITS1-5.8S-ITS2		Alpha-tubulin gene	
	Accession No.	Length in bp	Accession No.	Length in bp	Accession No.	Length in bp
*Anteholosticha gracilis*	-	-	-	-	GQ258104	1074
*Anteholosticha manca*	-	-	-	-	GQ258111	1074
*Anteholosticha parawarreni*	FJ870074	1784	-	-	-	-
*Anteholosticha eigneri*	-	-	-	-	GQ258105	1074
*Apokeronopsis bergeri*	-	-	-	-	GQ258112	1074
*Bergeriella ovata*	-	-	GQ246479	552	GQ258113	1074
*Epiclintes auricularis auricularis*	-	-	-	-	GQ262001	1074
*Epiclintes auricularis rarisetus*	-	-	GQ246480	483	-	-
*Holosticha diademata*	-	-	-	-	GQ258106	1074
*Metaurostylopsis cheni*	-	-	GQ246481	537	GQ258114	1074
*Nothoholosticha fasciola*	-	-	-	-	GQ258107	1074
*Parabirojimia multinucleata*	-	-	GQ246483	517	GQ258108	1074
*Psammomitra retractilis*	-	-	GQ246483	478	-	-
*Pseudoamphisiella quadrinucleata*	-	-	GQ246484	483	GQ258109	1074
*Pseudokeronopsis carnea*	-	-	-	-	GQ258110	1074
*Pseudourostyla cristata*	-	-	GQ246486	504	GQ258115	1074
*Thigmokeronopsis stoecki*	-	-	GQ246485	480	-	-

The SSU rRNA gene had the most characters (1,635 bp unambiguously aligned), followed by alpha-tubulin (1,071 bp), then ITS1-5.8S-ITS2 (427 bp) for the 14-taxon datasets. The nucleotide sequences of all three genes among 14 urostylids share similarities of 90.59–99.26%, 52.77–94.03%, and 77.40–91.96%, respectively (Tables S1, S2). It is noteworthy that alpha-tubulin amino acid sequences share similarities of 97.13–100.00% (Table S2), so phylogenetic trees were constructed using alpha-tubulin nucleotide sequences instead of amino acid sequences in our analyses.

**Figure 1:**
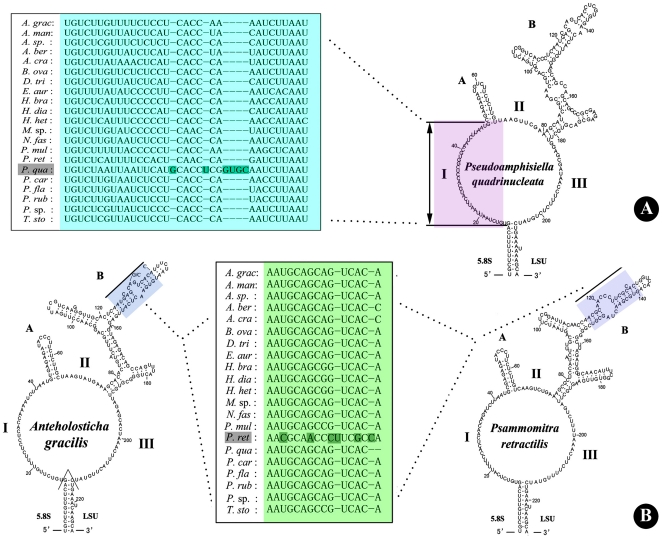
Secondary structures of the internal transcribed spacer 2 (ITS2) RNA transcript of three representative urostylid species (Viz. *Anteholosticha gracilis*, *Psammomitra retractilis*, *Pseudoamphisiella quadrinucleata*), and sequence alignments of two unique regions. The diagrams illustrate the two helices, labeled A and B, present in the class Spirotrichea [41]. Three parts of the biggest loop are labeled I, II and III, respectively. Lines beside *A. gracilis* and *Psammomitra retractilis* denote the region of greatest primary sequence conservation for class Spirotrichea [41]. Unique nucleotide sites are highlighted.

Comparisons of the ITS2 region sequences as well as secondary structures (Figure S1) show that there are two unique regions for *Pseudoamphisiella quadrinucleata*, and one for *Psammomitra retractilis*. As shown in Fig. 1, the main loop is divided into three parts (viz. I, II, and III) by Helix A and B, and there are 37 nucleotides in part I of *Pseudoamphisiella*, whereas there are only 31 ones in other species (Fig. 1A). Helix A in *Pseudoamphisiella* contains 19 nucleotides, whereas that of other species is constantly composed of 20 nucleotides. This is caused by one nucleotide deletion in the terminal loop of Helix A for *Pseudoamphisiella* (data not shown). Previous investigations [41]–[43] showed that for spirotricheans, 11 out of 12 paired nucleotides were identical in the labeled 15 nucleotides stretch of Helix A. However, our current analysis (Fig. 1B) indicates that *Psammomitra* has rather different sequences and secondary structure in this region.

ILD tests for all combined datasets (viz. Datasets 4, 5, 9–11) show that most of the partitioned datasets contain conflicting signal (*P* = 0.001), with only Dataset 9 being congruent (*P* = 0.256). In an attempt to further clarify the incongruence, each taxon was deleted in turn to determine if one or a few taxa were particularly problematic. However, in no dataset did this approach indicate that conflict is potentially caused by a specific taxon (Table 2).

**Table 2:** Results of the ILD Test of Congruence of Datasets.

Taxa	Dataset 5	Dataset 9	Dataset 10	Dataset 11
All taxa	0.001	**0.256**	0.001	0.001
Excluded:				
*Anteholosticha eigneri*	0.001	**0.969**	0.001	0.001
*Anteholosticha gracilis*	0.001	**0.178**	0.001	0.001
*Anteholosticha manca*	0.001	**0.151**	0.001	0.001
*Apokeronopsis bergeri*	0.001	**0.171**	0.001	0.001
*Bergeriella ovata*	0.001	**0.404**	0.001	0.001
*Holosticha diademata*	0.001	**0.601**	0.001	0.001
*Metaurostylopsis cheni*	0.001	**0.147**	0.001	0.001
*Nothoholosticha fasciola*	0.001	**0.109**	0.001	0.001
*Parabirojimia multinucleata*	0.001	**0.998**	0.001	0.001
*Psammomitra retractilis*	0.001	**0.312**	0.001	0.001
*Pseudoamphisiella quadrinucleata*	0.001	**0.122**	0.001	0.001
*Pseudokeronopsis carnea*	0.001	**0.231**	0.001	0.001
*Pseudourostyla cristata*	0.001	**0.108**	0.001	0.001
*Thigmokeronopsis stoecki*	0.001	**0.211**	0.001	0.001

NOTE.-Significant *P* values≥0.05 in bold.

### Phylogenetic Analyses Inferred from Dataset 1 (SSU rRNA, 89 Taxa)

**Figure 2:**
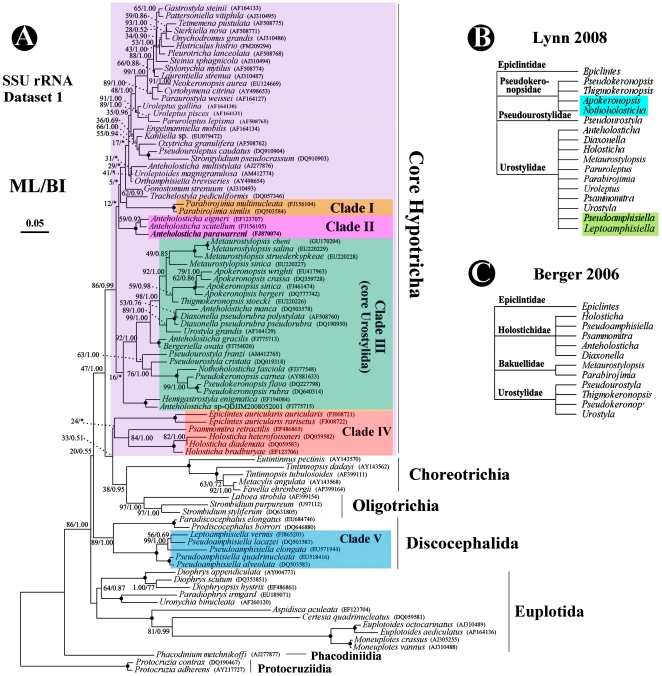
Phylogeny of the class Spirotrichea inferred by ML of SSU rRNA gene sequences (A), and two systems (B, C). A. Urostylids are labeled in colours. Species newly sequenced in the present study is shown in bold type. BP for ML tree and PP for BI tree are given near nodes, respectively. Asterisks show different node topologies between BI and ML trees. Fully supported (100%/1.00) branches are marked with solid circles. The scale bar corresponds to 5 substitutions per 100 nucleotide positions; black dot marks the genus *Hemigastrostyla* which is a non-urostylid. B. System of Lynn [1] containing only sequenced urostylid genera, with several highlighted genera not included by Lynn [1]. C. System of Berger [9] containing only sequenced urostylid genera.

In our analyses (Fig. 2A), the outgroup Protocruziidia is followed by Phacodiniidia and Euplotida, then the sister clade forming by Oligotrichia and Choreotrichia. Hypotricha seems to be paraphyletic: most species group together, and others cluster with Oligotrichia, Choreotrichia, and the core discocephalids, respectively.

Though *Uroleptus* and *Paruroleptus* are assigned into the family Urostylidae according to Lynn [1], they are undoubtly classified out of the order Urostylida in our SSU rRNA gene tees (Fig. 2), which is congruent with previous investigations [9], [12], [23], [24], [44]. Considering exclusion of these two genera from the order Urostylida, all available SSU rRNA gene sequences of urostylids were included in our phylogenetic analyses, and they refer to 15 genera representing all four urostylid families (sensu Lynn [1]) and four unclassified genera (Fig. 2). In both analyses, the order appears to be always paraphyletic, and species fall into six clades, except for *Anteholosticha multistilata*, the position of which is unresolved. Clade I consists of two *Parabirojimia* species (family Urostylidae), which group with *Trachelostyla*, a non-urostylid genus. Clade II consists of three *Anteholosticha* species. Clade III is the “core” urostylid clade, and it is composed of seven genera which belong to the family Urostylidae (viz. *Metaurostylopsis*, *Urostyla*, *Diaxonella* and *Anteholosticha*), two genera of the family Pseudokeronopsidae (*Pseudokeronopsis*, *Thigmokeronopsis*), one genus of the family Pseudourostylidae (*Pseudourostyla*), three unclassified urostylid genera (*Apokeronopsis*, *Bergeriella* and *Nothoholosticha*), and the non-urostylid genus *Hemigastrostyla* [1], [45]. Clade IV has a closer relationship with Oligotrichia and Choreotrichia than with other urostylids, and consists of two genera of the family Holostichidae (viz. *Holosticha* and *Psammomitra*), and the type genus of the family Epiclintidae, *Epiclintes*. Clade V falls into the order Discocephalida, and consists of *Pseudoamphisiella* (family Holostichidae) and the unclassified genus *Leptoamphisiella* (see Discussion). Among the four urostylid families, the Epiclintidae is monotypic whereas the other three are multi-generic and paraphyletic. All species of Pseukeronopsidae and Pseudourostylidae fall into Clade III, and urostylid species appear in all six clades. Among 15 sequenced urostylid genera, species of *Anteholosticha* are the most diverse and representatives could be found in both Clades II and V. Of the other 14 genera, none have representatives in more than one clade.

### Phylogenetic Analyses Inferred from Dataset 2 (ITS1-5.8S-ITS2, 31 Taxa), Dataset 3 (Alpha-Tubulin, 26 Taxa) and Dataset 4 (Three-Gene Combined, 25 Taxa)

**Figure 3 pone-0017471-g003:**
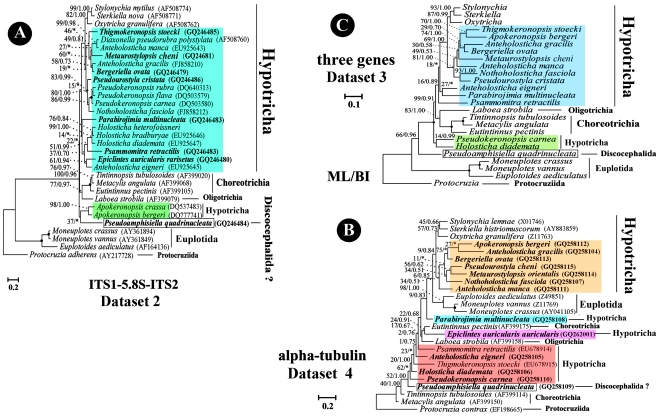
Phylogeny of the class Spirotrichea inferred by ML of Datasets 2–4 (A–C). Urostylids are labeled in colours. Species newly sequenced in the present study are shown in bold type. BP for ML tree and PP for BI tree are given near nodes, respectively. Asterisks show different node topologies between BI and ML trees. Fully supported (100%/1.00) branches are marked with solid circles. The scale bar corresponds to 10/20 substitutions per 100 nucleotide positions.

As revealed in trees based on Dataset 1 (Fig. 2A), analyses inferred from Datasets 2 and 4 (Fig. 3A and 3C) also indicate that: (1) the outgroup Protocruziidia is followed by Euplotida, Oligotrichia, Choreotrichia; (2) Hypotricha is separated into several clades; (3) the core urostylid group contains only genera/species of Clade IV derived from Dataset 1 (Fig. 2A), namely *Anteholosticha gracilis*, *A. manca*, *Bergeriella*, *Diaxonella* (absent from Dataset 4), *Metaurostylopsis*, *Thigmokeronopsis*, *Apokeronopsis* (which does not cluster with this group in trees based on Dataset 2), *Pseudokeronopsis*, *Pseudourostyla*, and *Nothoholosticha*; (4) *Pseudoamphisiella* is rather distant from other urostylids in Datasets 2, 4. However, the cluster pattern of species outside the core urostylid group is rather different among trees based on Datasets 1, 2, and 4.

In analyses inferred from Dataset 3, the subclass Protocruziidia branches at the deepest level, however, compared to trees based on Datasets 1, 2, and 4, the clade comprising the euplotids is more closely related to the “core” Hypotricha. The monophyly of Choreotrichia is rejected. In addition, *Thigmokeronopsis* and *Pseudokeronopsis*, which belong to the core urostylids in analyses based on Datasets 1, 2, and 4, fall outside the core Urostylida.

### Comparison of Phylogenetic Analyses Inferred from 14-Taxa Datasets

**Figure 4:**
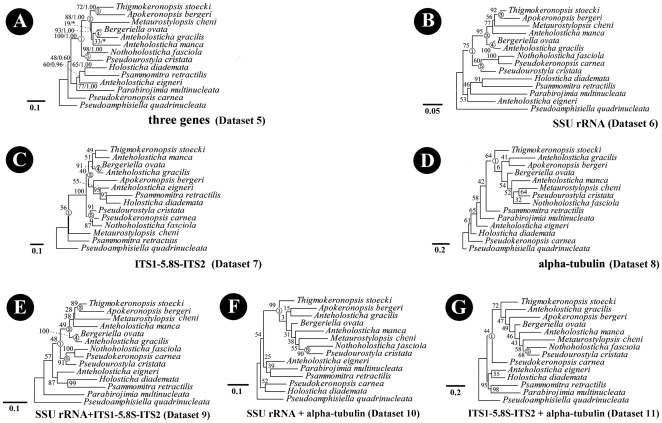
Phylogeny of the class Spirotrichea inferred by ML of 14-taxa Datasets 5–11 (A–G). The scale bar corresponds to 5/10 substitutions per 100 nucleotide positions. Circled numbers refer to node numbers in PABA approach.

ML tree topologies inferred from seven 14-taxa datasets (Datasets 5–11) (Fig. 4) were not identical to each other. However, as revealed by trees based on Datasets 1–4, these analyses also strongly indicate that: (1) *Pseudoamphisiella* should be excluded from urostylids, and; (2) the core urostylid group contains *Anteholosticha manca*, *A. gracilis*, *Bergeriella*, *Metaurostylopsis*, *Thigmokeronopsis*, *Apokeronopsis*, *Pseudokeronopsis*, *Pseudourostyla* and *Nothoholosticha*.

Using the S-H approach, out of 42 possible comparisons, 15 ones result in a *P* value above 0.05, signaling that congruence is not rejected, whereas 27 comparisons reject congruence (*P*<0.05) (Table 3). Dataset 5 rejects all topologies inferred from other 14-taxa datasets, however, two topologies among them are not totally rejected. Conversely, topology based on Dataset 5 is only rejected by Dataset 7. Interestingly, all topologies obtained by datasets including alpha-tubulin are accepted by other datasets also including alpha-tubulin (Fig. 4A, D, F, G), but are rejected by all other datasets (Fig. 4B, C, E).

**Table 3 pone-0017471-t003:** Results of the SH Test of Congruence of Datasets.

Datasets	Topology (ML)						
	Dataset 5	Dataset 6	Dataset 7	Dataset 8	Dataset 9	Dataset 10	Dataset 11
Dataset 5	–	**0.140**	<0.001	**0.036**	**0.212**	**0.150**	**0.275**
Dataset 6	0.002	–	<0.001	<0.001	**0.669**	<0.001	<0.001
Dataset 7	<0.001	0.001	–	<0.001	**0.053**	<0.001	<0.001
Dataset 8	<0.001	<0.001	<0.001	–	<0.001	**0.415**	**0.355**
Dataset 9	<0.001	**0.442**	**0.233**	<0.001	–	<0.001	<0.001
Dataset 10	0.038	0.005	<0.001	**0.285**	0.003	–	**0.702**
Dataset 11	0.049	<0.001	<0.001	**0.603**	0.004	**0.508**	–

NOTE.-Significant *P* values≥0.05 in bold.

Five, three, one, five, two and two of five nodes selected based on 14-taxa three-gene combined datasets could be found in trees inferred from Datasets 6–11, respectively (Fig. 4B–G). For Node 1, the addition of ITS1-5.8S-ITS2 region data causes the bootstrap values to decrease (Table S3). For Nodes 2–4, the addition of alpha-tubulin gene data does the same thing (Tables S4, S5, S6). By contrast, the addition of SSU rRNA gene data always increases the support values (Tables S3, S4, S5, S6, S7). Considering all five nodes, the PABA approach also shows that bootstrap values tend to increase as more data or data partitions are added except when alpha-tubulin gene data is added as the second partition (Table 4).

**Table 4:** Alteration of Bootstrap Support δ to Nodes in Fig. 4 as Gene Partitions Are Added.

<?tvs=-2.5pt?>Nodes	BP value of Dataset 5	Gene partitions					
		Alpha-tubulin		ITS1-5.8S-ITS2		SSU rRNA	
		2nd	3rd	2nd	3rd	2nd	3rd
1	100	6	52	−24	0	14	56
2	88	−58	39	−13	88	5	88
3	72	−46	−17	−2	72	45	72
4	93	−96	−7	0	93	5	93
5	98	4	7	50	8	45	30
Average over all nodes		−38	15	2	52	23	68

## Discussion

This study represents one of the few attempts to reconstruct generic-level relationships within Urostylida with molecular characters from multiple genes, and the only phylogenetic analysis that includes all four urostylid families. Though the phylogenetic results based on different datasets are mixed, and support values for some nodes are not high (Fig. 2–4), some conclusions could be drawn following by comparison between our phylogenetic trees and system of Lynn [1].

### The Current Status of the Phylogenetic Relationships within the Order Urostylida

Recent molecular phylogenetic investigations (for example, [ 4], [12], [22]–[27]), as well as the current work based on both single gene (Datasets 1–3, Fig. 2A, 3A, B) and multiple genes (Dataset 4, Fig. 3C) shows that the urostylid assemblage is not monophyletic and thus raises serious challenges to the classification of the order Urostylida [1], [8], [9], [14]–[16], [18], [21]. This is consistent with the conclusion that there is a considerable amount of convergence in urostylid morphology [9] which brings into question current classification scheme [1]. In the present work, several datasets, with SSU rRNA, alpha-tubulin and ITS1-5.8S-ITS2 gene/region sequences for all known urostylid genera, were used in order to re-evaluate phylogenetic relationships within this assemblage and to make a comparison between molecular phylogeny and the system of Lynn [1] which is mainly based on morphological/morphogenetic data.

### Classification of Four Unclassified Genera

The systematic positions of four recently reported genera, namely *Bergeriella*, *Leptoamphisiella*, *Apokeronopsis* and *Nothoholosticha,* were not included in any of updated systems although they were putatively assigned to the order Urostylida based on either morphological/morphogenetic or molecular information in the original descriptions [10], [11], [46], [47]. Among them, a new family, Bergeriellidae, was erected for the type genus *Bergeriella* [11]. In the present investigation, *Bergeriella* always falls into core urostylid group in all the trees, and is not closely related to any of the four urostylid families [1] (Fig. 2–4). Thus, according to both molecular and morphological/morphogenetic data, all the evidence supports the conclusion that *Bergeriella* should represent a distinct family within the order Urostylida [11].

The results presented here show that the genus *Leptoamphisiella* is most related to *Pseudoamphisiella*, the type genus of the family Pseudoamphisiellidae, which is, however, assigned to the family Urostylidae in Lynn's system [1]. Our analyses firmly support the conclusion that this family should be excluded from the order Urostylida, but rather belongs to a group of its own which clusters to the well-known discocephalids [23], [48].

Both *Apokeronopsis* and *Nothoholosticha* are confirmed as true urostylids belonging to the family Pseudokeronopsidae [10], [47].

### Classification of the Family Urostylidae

Nine genera included in our analyses (viz. *Anteholosticha*, *Diaxonella*, *Holosticha*, *Metaurostylopsis*, *Parabirojimia*, *Psammomitra*, *Urostyla*, *Pseudoamphisiella* and *Leptoamphisiella*), all of which are assigned to the family Urostylidae in Lynn's [1] system, are distributed among Clades I–VI in the present analysis (Fig. 2A).

As revealed in previous molecular and morphological investigations [1], [4], [9], [12], [24] and in our SSU rRNA gene trees (Fig. 2A), *Uroleptus* and *Paruroleptus* should be removed from the urostylid family Urostylidae [for details see discussion in 24] to the non-urostylid family Uroleptidae [44]. Similarly, *Pseudoamphisiella* and *Leptoamphisiella*, two urostylid genera according to Lynn [1], should be placed in the suborder Discocephalina since they consistently cluster with Discocephalina (Fig. 2A). This is consistent with the results of previous studies based on molecular data [6], [23], [48], and supports the findings that some morphological/morphogenetic features of these genera, e.g. the cirri of the midventral complex are not arranged in the zig-zag pattern, and the general developmental process of the ciliary structure, are more similar to those of discocephalines than urostylids [49].

The phylogenetic position of *Parabirojimia* is slightly variable according to different datasets, however, it always falls outside of the “core” urostylid group and does not have a robust relationship with any other typical urostylids (Fig. 2–4). Considering the extremely unusual mode of development of the cortical structure during morphogenesis, especially the formation of the somatic ciliature, e.g. the transverse cirri, the right marginal rows, etc. [50], it is reasonable to assign this genus/family to its own group, that is the suborder Parabirojimina, as suggested by Yi et al. [23].

The genus *Metaurostylopsis* is only included in three systems [1], [9], [51]. Among those genera included in the present investigation, Shi et al. [51] considered that *Metaurostylopsis* has a close relationship with *Urostyla* and *Pseudourostyla*, Berger [9] placed it together with *Parabirojimia* in family Bakuellidae, and five other (non-sequenced) genera, whereas Lynn [1] suggested that *Metaurostylopsis* could be related to *Anteholosticha*, *Holosticha*, *Diaxonella*, *Parabirojimia*, *Psammomitra*, *Pseudoamphisiella*, and *Uroleptus*. However, among these hypotheses, only the sister relationship between *Metaurostylopsis* and *Pseudourostyla* is hinted by Dataset 3 (Fig. 3B), indicating that none of the assignments of *Metaurostylopsis* in these three systems are reasonable.

As noted by Berger [9], the systematic position of *Diaxonella* is complicated since the type species, *D. pseudorubra*, has been repeatedly reported under different generic and specific names (for example, [ 9], [18], [52]–[56]). This genus has only been included in two systems [1], [9], since it was established by Jankowski [20]. Based on the redescription of *D. pseudorubra* (as *D. trimarginata* by Shao et al. [55]), it was assigned to the family Pseudourostylidae, thus as an urostylid species. This report also included a description of morphogenesis and the unusual mode of formation of left marginal rows, which has been reported in only another hypotrich genus, that is, *Pseudourostyla*. However, the present and previous molecular investigations [4], [12], [22], [23], [27], [40], [57], [58] did not recover a close relationship between *Diaxonella* and *Pseudourostyla*, thus supporting Berger's [9] hypothesis that this unusual morphogenetic process is very likely a result of convergent evolution and should not be regarded as a family level character as suggested by Eigner and Foissner [8]. In addition, the placement of *Diaxonella* in family Holostichidae (sensu Berger [9]) is also clearly rejected by the molecular data in both the present and previous investigations [4], [12], [22], [23], [27], [40]. This is consistent with the morphological finding that *Diaxonella* has more than two marginal rows, and is hence rather different from other holostichid genera (sensu Berger [9]). According to Lynn [1], *Diaxonella* should be assigned into the family Urostylidae. However, only the connection between this genus and *Urostyla*, and *Anteholosticha manca* is accepted in the present work (Fig. 2A, 3A) and previous investigations [4], [12], [22], [23], [27], [40], [57], [58]. All this evidence indicates that *Diaxonella* is undoubtedly an urostylid, however its family-level assignment in both Berger's [9] and Lynn's [1] systems is highly questionable and needs to be re-evaluated.

Of the final four genera, viz. *Holosticha*, *Psammomitra*, *Urostyla* and *Anteholosticha* which are also assigned to the family Urostylidae by Lynn [1], the first three are located in two separate clades in our trees (Fig. 2–4). The relationship between *Holosticha* and *Psammomitra* hypothesized by Lynn [1] and Berger [9] was confirmed by both previous [22] and present analyses except in trees based on single-gene datasets and in those based on datasets containing alpha-tubulin information with two genes combined (Fig. 2–4). By contrast, the genus *Anteholosticha* appears to be heterogeneous and highly divergent, with species falling into different clades in all our trees (Fig. 2–4). In addition, distinct from other genera, seven *Anteholosticha* species share no unique nucleotides at semi-conserved, parsimony-information sites in the alignment of SSU rRNA gene sequences. These findings indicate that *Anteholosticha* is probably a convergent assemblage of species as predicted also by Berger [9], [59] and a revision of this genus is urgently needed.

In summary, the family Urostylidae (sensu Lynn [1]) seems to be a huge “melting pot” containing over 24 nominal genera, the monophyly of which is strongly rejected by the present analyses (Fig. 2). Currently, a complete re-arrangement for its classification remains impossible partly because molecular information is lacking for too many taxa. Nevertheless, the following conclusions can be drawn based on our analyses: 1) as revealed in previous investigations [9], [12], [22]–[24], [44], *Parabirojimia*, *Psammomitra*, *Pseudoamphisiella*, *Leptoamphisiella*, *Uroleptus* and *Paruroleptus* should be removed from this family and the last four genera are not even members of the order Urostylida. 2) *Holosticha* should also be excluded from this family; 3) *Diaxonella* and *Urostyla*/*Parabirojimia* respectively might represent two isolated families; 4) the genus *Anteholosticha* is extremely diverse, polyphyletic and should be revised when more information becomes available.

### Classification of the Family Pseudokeronopsidae

Two of the six genera in the family Pseudokeronopsidae (sensu Lynn [1]), viz. *Pseudokeronopsis* and *Thigmokeronopsis*, and another two genera which should be included in this family, viz. *Apokeronopsis* and *Nothoholosticha*, group consistently into two clades in SSU rRNA trees (Fig. 2A, 4B), and into more then two clades in other trees (Fig. 3, 4A, C–G). Thus, all of these analyses reject the monophyly of this family.

Berger [9] synonymised *Apokeronopsis begeri* as *Thigmokeronopsis crassa*, due to the genus *Apokeronopsis* was not erected then. However, phylogenetic trees based on Datasets 2, 3, 7, 8, 10, and 11, none of which contain SSU rRNA gene sequences except Dataset 10, failed to recover a close relationship between these two genera (Fig. 3A, B, 4C, D, F, G ), although they did group together in other trees including SSU rRNA gene (Fig. 2A, 3C, 4A, B, E), which indicates that the connecting of *Apokeronopsis* with *Thigmokeronopsis* is probably due to inclusion of SSU rRNA. Considering the separation of these two genera is supported by some morphological/morphogenetic data, for example, presence or absence of thigmotactic cirri and the fusion pattern of macronuclear segments prior to division [47], [60], the distinction of both genera is reliable but their systematic positions remain unresolved.

Although *Thigmokeronopsis* and *Pseudokeronopsis* are placed into the family Pseudokeronopsidae by most investigators e.g., [ 1], [8], [9,14], a sister relationship between these two genera is not revealed in any of our trees (Fig. 2–4), nor in previous molecular phylogenetic analyses [11], [12], [22], [40]. The relationship between *Pseudokeronopsis* and *Nothoholosticha* is clearly supported both by morphological (viz. midventral pairs arranged in a zig-zag pattern, distinctly fewer transverse cirri than midventral cirral pairs, and one marginal row on each side of the body) and phylogenetic trees based on SSU rRNA gene and ITS-5.8S-ITS2 region sequences [Figs 2A, 3A, 4B, C, E in present investigation,] [40]. However, no close relationship is recovered in trees containing alpha-tubulin gene sequences (Fig. 3B, C, 4A, D, F, G).

As a primary conclusion, it appears that the family Pseudokeronopsidae is not monophyletic although most of its members almost certainly belong to the core portion of urostylids. Very likely, some or most pseudokeronopsids should be transferred to the family Urostylidae, although a taxonomic revision of this group must await further data.

### Classification of Acaudalia and the Family Pseudourostylidae

The family Pseudourostylidae comprises three genera, *Hemicycliostyla*, *Trichotaxis* and *Pseudourostyla* (sensu Lynn [1]). SSU rRNA gene sequence data is available for only two pseudourostylids, viz. *Pseudourostyla franzi* and *P. cristata*. This classification is consistent with that of Berger [9]. In our SSU rRNA gene trees, two *Pseudourostyla* species group with the *Pseudokeronopsis-Nothoholosticha* cluster, which is a sister group to other typical urostylids, e.g. *Anteholosticha*, *Metaurostylopsis*, *Apokeronopsis* etc. (Fig. 2A). And Chen et al. [61] observed that, *Pseudourostyla* is morphologically similar to *Urostyla* and *Metaurostylopsis*, albeit with some minor morphological and morphogenetic differences. The latter two, however, were assigned to the family Urostylidae by Lynn [1]. According to Berger [9], *Pseudourostyla*, *Thigmokeronopsis*, *Apokeronopsis* (syn. *Thigmokeronopsis*), and *Pseudokeronopsis* are included in the unranked higher taxon Acaudalia Berger, 2006. The monophyly of Acaudalia, however, is not recovered in any of our trees (Fig. 2–4), and is rejected by AU tests (*P*<0.05), which is consistent with several previous reports [12], [22], [27], [40], [58], although close relationships between *Thigmokeronopsis* and *Apokeronopsis*, and between *Pseudourostyla* and *Pseudokeronopsis*, were recovered in some trees (Fig. 2–4).

### Classification of the Family Epiclintidae

The family Epiclintidae (Wicklow & Borrow 1990) contains two genera, viz. *Epiclintes* and *Eschaneustyla* [1], [9]. Due to the absence of gene sequences for *Eschaneustyla*, however, the evolutionary relationships of these genera cannot be evaluated using molecular data.

The phylogenetic position of *Epiclintes* is subject to a long and ongoing dispute due to its unusual cirral pattern. As referred in Berger [9], it has been historically assigned to the families Oxytrichidae [20], [62]–[64], Urostylidae [64]–[67], Amphisiellidae [68], Keronidae [15], [69]–[73], Spirofilidae [74], [75], or as *incertae sedis* within the order Stichotrichida [76]. Based on morphological and ultrastructural specializations, Wicklow and Borror [77] established the family Epiclintidae for this genus, and supposed that *Epiclintes* is a specialized descendent from *Kahliella*-like stichotrichines. In a recent study, Hu et al. [57] rejected the placement of *Epiclintes* in the families Oxytrichidae, Amphisiellidae, and Spirofilidae, or in the order Stichotrichida. Furthermore, several morphological and morphogenetic features of *Epiclintes* were found to be inconsistent with those of urostylids, including: (1) many oblique ventral rows originating from cirral anlagen but no zigzagic pattern formed, (2) a short row of frontal cirri deriving from UM-anlage, (3) partial replacement of the old adoral zone, (4) de novo formation of the oral primordium, the anlagen for marginal rows and dorsal kineties [57]. The results of the present study are consistent with these findings and also reject a close relationship between *Epiclintes* and *Kahliella* (Fig. 2A). As a basal clade, it branches deeply from the assemblage of three *Holosticha* and one *Psammomitra* species. Thus, all the available evidence supports the separation of the Epiclintidae at family/suborder level as suggested previously [57], [77], [78].

### Congruence/Incongruence among Different 14-Taxa Datasets

Seven phylogenies based on seven different datasets (Datasets 5-11) with same taxa were topologically incongruent, however, a “core” urostylid group is revealed in each tree (Fig. 4). *Anteholosticha manca*, *A. gracilis*, *Bergeriella*, *Metaurostylopsis*, *Thigmokeronopsis*, *Nothoholosticha* and *Pseudourostyla*, always fall into this core group, whereas *Pseudokeronopsis*, *Holosticha* and *Psammomitra* only cluster within this group in some Datasets (Fig. 4). Among the core group, five nodes are chosen to test congruence among partitions (Fig. 4).

In these seven 14-taxa analyses, ILD, S-H and PABA tests were used to detect congruence/incongruence among different partitions (Tables 2–4, Tables S3, S4, S5, S6, S7). The ILD test fails to show congruence among most datasets, and only Dataset 9 is suggested to be combined (Table 2). By contrast, the S-H test shows that none of the tree topologies based on combined datasets (Datasets 5, 9, 10, 11) are totally rejected by all other datasets (Table 3). Furthermore, the PABA approach revealed that, apart from the addition of alpha-tubulin gene as the second partition, all additions of partitions increase average BP over all five selected nodes (Table 4). This is consistent with previous investigations [37], [79], [80], the ILD test appears to be too conservative, and should only used as a measure of heterogeneity between gene partitions rather than a measure for a combinability test. The ILD test indicates that SSU rRNA and ITS1-5.8S-ITS2 are congruent, and that alpha-tubulin is incongruent with them, whereas the S-H tests fail to pinpoint the cause of conflict.

For the PABA approach, the mean bootstrap alteration values in Table 4 suggest that in general the SSU rRNA gene contributed the most signal, followed by ITS1-5.8S-ITS2, and then alpha-tubulin. This is consistent with results of all five separated nodes, which shows that all partitions increase BP for Node 5 (Table S7), whereas ITS1-5.8S-ITS2 decrease BP for Node 1 (Table S3), and alpha-tubulin decrease BP for the other three nodes (Tables S4, S5, S6). This is reasonable, considering that the SSU rRNA and ITS1-5.8S-ITS2 genes locate near each other, and SSU rRNA possesses most characters in our analyses.

## Materials and Methods

### Selection and Identification of Ciliates

The taxa in this study were selected to represent the morphological and morphogenetic diversity of Urostylida. Although the current taxon sampling does not cover all genera in Urostylida, representative taxa for each family were included.

*Bergeriella ovata* (Liu et al. 2010), *Parabirojimia multinucleate* (Chen et al. 2010), and *Pseudoamphisiella quadrinucleata* (Shen et al. 2008) were collected from the coast near Guangzhou, southern China (22°42′N; 114°32′E). Other species and strains were collected from the coast near Qingdao, northern China (36°08′N; 120°43′E). All isolates were identified by the methods of Shao et al. [81] and Li et al. [10]. Terminology and systematic classification used in the current paper follow Berger [82] and Lynn [1], respectively.

### Extraction and Sequencing of DNA

Genomic DNA was extracted according to methods described in Yi et al. [22]. Eukaryotic universal A (5′-AACCTGGTTGATCCTGCC AGT-3′) or 82F (5′-GAAACTGCGAATGGCTC-3′) and Eukaryotic universal B (5′-TGATCCTTCTGCAGGTTCACCTAC-3′) primers were used for amplification of the SSU rRNA gene [83] by polymerase chain reaction (PCR). Cycling parameters for the SSU rRNA gene were as follows: 5 min initial denaturation (94°C), followed by 35 cycles of 1 min at 95°C, 1 min 30 s at 56°C, and 2 min at 72°C, with a final extension of 15 min at 72°C. A fragment of approximately 500 bp containing the ITS1, 5.8S ribosomal gene, and ITS2 was amplified using primers ITS-F (5′-GTAGGTGAACCTGCGGAAGGATCATTA-3′) and ITS-R (5′-TACTGATATGCTTAAGTTCAGCGG-3′) [84], with the following cycling parameters: 5 min initial denaturation (94°C), followed by 35 cycles of 30 s at 95°C, 1 min at 56°C, and 1 min at 72°C, with a final extension of 15 min at 72°C. A fragment of approximately 1,000 bp comprising part of the alpha-tubulin gene was amplified using ciliate-specific primers Tub-1 (5′-AAGGCTCTCTTGGCGTACAT-3′) and Tub-2 (5′-TGATGCCTTCAACACCTTCTT-3′) [22]. Cycling parameters were as follows: 5 min initial denaturation (94°C), 35 cycles of 30 s at 94°C, 1 min at 56°C, and 1.5 min at 72°C, with a final extension of 15 min at 72°C. Purified PCR product of appropriate size was inserted into the pUCm-T vector (Shanghai Sangon Biological Engineering & Technical Service Company, China) and sequenced at the Invitrogen sequencing facility in Shanghai, China.

### Databases Selection

Eight datasets were evaluated in our analyses: (1) SSU rRNA gene sequences including all available urostylid sequences plus some other spirotricheans (89 sequences in total); (2) ITS1-5.8S-ITS2 region sequences including all available urostylid sequences plus some other spirotricheans (31 sequences in total); (3) alpha-tubulin gene sequences including all available urostylid sequences plus some other spirotricheans (26 sequences in total); (4) three-gene combined dataset including all spirotrichean species available, and *Protocruzia adherens*, *Stylonychia mytilus* and *Sterkiella nova* for SSU rRNA and ITS1-5.8S-ITS2, and *Protocruzia contrax*, *Stylonychia lemnae* and *Sterkiella cavicola* for alpha-tubulin (25 sequences in total); (5) three-gene combined dataset including all available urostylid species (14 sequences in total); (6) SSU rRNA gene sequences including all taxa in Dataset 5; (7) ITS1-5.8S-ITS2 region sequences including all taxa in Dataset 5; (8) alpha-tubulin gene sequences including all taxa in Dataset 5; (9) two-gene combined dataset composed of Datasets 6 and 7; (10) two-gene combined dataset composed of Datasets 6 and 8; (11) two-gene combined dataset composed of Datasets 7 and 8.

### Secondary Structure Prediction and ITS2 Sequence Alignment

The default settings of the mfold website (http://frontend.bioinfo.rpi.edu/applications/mfold) [85] were used to produce the secondary structure and sequence in dot-bracket structural format of ITS2 RNA transcripts. The structures were edited for aesthetic purposes with RnaViz 2.0 [86].

The ITS2 sequences with the secondary structure format were aligned using the MARNA web server (http://biwww2.informatik.uni-freiburg.de/Software/MARNA/index.html) [87], based on both the primary and secondary structures.

### Phylogenetic Analyses

Sequences (except for ITS2 sequences) were aligned using the ClustalW implemented in Bioedit 7.0.0 [88] and further modified manually using Bioedit.

The final alignment of Dataset 1 included 1,607 positions, and the alignment is available from the authors upon request. A Bayesian inference (BI) analysis was performed with MrBayes 3.1.2 [89] using the GTR+I+G model selected by MrModeltest 2 [90] under the AIC criterion. Markov chain Monte Carlo (MCMC) simulations were run with two sets of four chains using the default settings: chain length 2,000,000 generations, with trees sampled every 100 generations. The first 5,000 trees were discarded as burn-in. The remaining trees were used to generate a consensus tree and to calculate the posterior probabilities (PP) of all branches using a majority-rule consensus approach. A Maximum Likelihood (ML) analysis was performed with PhyML V2.4.4 [91] using the GTR+G+I model selected under the AIC criterion by Modeltest v.3.7 [92]. The reliability of internal branches was assessed using a non-parametric bootstrap method with 1,000 replicates.

The following evolutionary models were selected by MrModeltest 2 for single datasets: GTR+I model for Datasets 2 and 7; GTR+I+G for Datasets 3, 6, and 8. Using these selected models, Bayesian trees for Datasets 2, 3, 4 were built as above. For Dataset 4, individual coding regions were treated as ‘unlinked’, so that separate parameter estimates as specified above were obtained for each gene partition for all runs.

The following evolutionary models were selected by Modeltest v.3.7 for different datasets: GTR+I model for Datasets 2 and 7; GTR+I+G for Datasets 3– 6, 8–11. Using these selected models, ML trees for Datasets 2–4 were constructed as above.

Phylogenetic trees were visualized with TreeView v1.6.6 [93] and MEGA 4 [94].

### Identifying of Congruence or Incongruence

Congruence of different data partitions (in this case genes) was tested with both the incongruence length difference (ILD) test [95] and Shimodaira-Hasegawa (S-H) test [96] as implemented in PAUP*4.0b. We excluded taxa with missing data in some gene partitions, and performed the ILD tests with Dataset 4 and Dataset 5, respectively. Six gene-by-gene comparisons were conducted based on 1,000 ILD replicates. In interpreting the results of ILD tests, recent studies have shown that the utility of the ILD test is limited as a measure of the incongruence among data partitions [79], [80]. Therefore, we used the ILD tests as a measure of heterogeneity between gene partitions and the results of ILD tests were not interpreted as a measure for a combinability test [79]. In the case of S-H tests, variance estimations of the difference in the likelihood values of given topologies to the best topology were used to test whether the topology produced by a given partition was accepted or rejected by different data partitions [80], [97]. Therefore, the major-rule consensus topologies obtained by the 7 different 14-taxon datasets were compared to each other based on each of these datasets using the S-H test. RELL approximations with 1,000 replicates and ML methods described above were conducted.

Because neither of these two approaches sufficiently described the source of possible incongruence and its influence in the dataset, the partition addition bootstrap alteration (PABA) approach [80] was used to evaluate the influence of combining genes on nodal support of “core Urostylida”, five nodes with high supports in three gene combined tree (Fig. 4A) were selected.

## Supporting Information

Figure S1 **ITS2 region secondary structures of 21 sequenced urostylid species.**
(TIF)

Table S1 **Evolutionary Similarities among Dataset 6 and 7, and Expressed as Percentages.**
(DOC)

Table S2 **Evolutionary Similarities among Dataset 9, and Expressed as Percentages.**
(DOC)

Table S3 **Alteration of Bootstrap Support δ Depending on the Order a Particular Partition Is Added Shown for Node 1 (See** **Figure 4** **).**
(DOC)

Table S4 **Alteration of Bootstrap Support δ Depending on the Order a Particular Partition Is Added Shown for Node 2 (See** **Fig. 4** **).**
(DOC)

Table S5 **Alteration of Bootstrap Support δ Depending on the Order a Particular Partition Is Added Shown for Node 3 (See** **Fig. 4** **).**
(DOC)

Table S6 **Alteration of Bootstrap Support δ Depending on the Order a Particular Partition Is Added Shown for Node 4 (See** **Fig. 4** **).**
(DOC)

Table S7 **Alteration of Bootstrap Support δ Depending on the Order a Particular Partition Is Added Shown for Node 5 (See** **Fig. 4** **).**
(DOC)
