# Supplementary figures and images for: Evolution of the Order Urostylida (Protozoa, Ciliophora): New Hypotheses Based on Multi-Gene Information and Identification of Localized Incongruence
Source: PLoS One. 2011 Mar 8;6(3):e17471. doi: 10.1371/journal.pone.0017471 (PMC3050893; doi:10.1371/journal.pone.0017471)

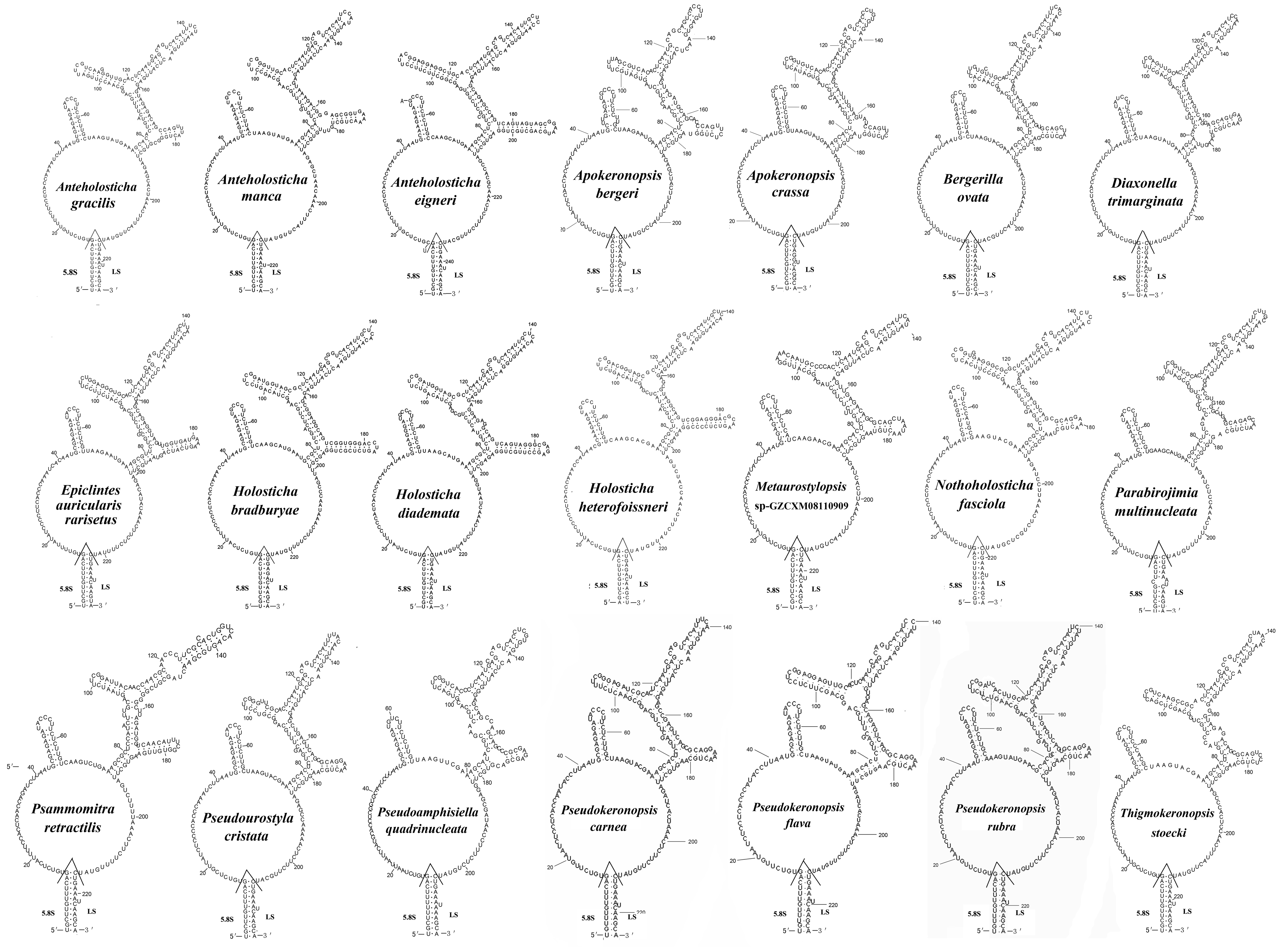

Supplement: Figure S1 — ITS2 region secondary structures of 21 sequenced urostylid species. (TIF) [file pone.0017471.s001.tif]
