# Supplementary material for: Evolution of the Order Urostylida (Protozoa, Ciliophora): New Hypotheses Based on Multi-Gene Information and Identification of Localized Incongruence
Source: PLoS One. 2011 Mar 8;6(3):e17471. doi: 10.1371/journal.pone.0017471 (PMC3050893; doi:10.1371/journal.pone.0017471)
Supplement: Table S1 — Evolutionary Similarities among Dataset 6 and 7, and Expressed as Percentages. (DOC) [file pone.0017471.s002.doc]

**Table S1:**

|  | 1 | 2 | 3 | 4 | 5 | 6 | 7 | 8 | 9 | 10 | 11 | 12 | 13 | 14 |
| --- | --- | --- | --- | --- | --- | --- | --- | --- | --- | --- | --- | --- | --- | --- |
| 1 *Anteholosticha eigneri* | -- | 79.59 | 75.88 | 75.39 | 80.29 | 80.54 | 64.43 | 77.08 | 68.63 | 80.53 | 74.47 | 62.95 | 82.28 | 76.54 |
| 2 *Anteholosticha gracilis* | 95.04 | -- | 83.40 | 75.45 | 94.03 | 74.42 | 72.32 | 78.65 | 67.53 | 81.97 | 71.09 | 67.05 | 83.46 | 82.92 |
| 3 *Anteholosticha manca* | 92.84 | 95.53 | -- | 74.20 | 84.09 | 70.00 | 68.25 | 75.31 | 65.47 | 79.59 | 67.83 | 61.94 | 80.42 | 82.55 |
| 4 *Apokeronopsis bergeri* | 93.34 | 96.33 | 94.98 | -- | 75.29 | 69.67 | 64.56 | 72.49 | 62.39 | 72.86 | 63.05 | 60.03 | 74.53 | 76.90 |
| 5 *Bergeriella ovata* | 94.04 | 99.26 | 95.92 | 96.52 | -- | 75.62 | 72.24 | 80.44 | 67.19 | 84.17 | 71.70 | 64.63 | 85.94 | 84.83 |
| 6 *Holosticha diademata* | 92.71 | 92.61 | 91.03 | 91.47 | 92.74 | -- | 64.46 | 74.28 | 62.14 | 74.61 | 73.93 | 55.75 | 73.70 | 73.83 |
| 7 *Metaurostylopsis* sp-QDCXM08060901 | 92.65 | 95.29 | 93.70 | 94.95 | 95.74 | 91.76 | -- | 68.76 | 72.34 | 69.44 | 55.60 | 68.30 | 71.52 | 73.97 |
| 8 *Nothoholosticha fasciola* | 93.55 | 93.96 | 92.27 | 93.33 | 94.28 | 91.97 | 92.93 | -- | 67.83 | 88.34 | 65.96 | 59.46 | 88.03 | 79.54 |
| 9 *Parabirojimia multinucleata* | 93.12 | 92.55 | 90.15 | 91.32 | 92.54 | 90.77 | 90.76 | 92.06 | -- | 68.57 | 55.72 | 68.38 | 71.68 | 67.13 |
| 10 *Pseudokeronopsis carnea* | 93.93 | 94.27 | 93.08 | 93.71 | 94.67 | 93.11 | 93.58 | 97.12 | 92.03 | -- | 69.68 | 64.79 | 89.21 | 77.52 |
| 11 *Psammomitra retractilis* | 92.38 | 92.76 | 90.96 | 91.40 | 92.61 | 92.29 | 91.63 | 92.24 | 91.08 | 92.28 | -- | 52.77 | 69.32 | 69.09 |
| 12 *Pseudoamphisiella quadrinucleata* | 91.52 | 91.93 | 90.59 | 92.10 | 92.20 | 89.83 | 91.56 | 90.59 | 89.83 | 90.91 | 90.22 | -- | 66.14 | 61.88 |
| 13 *Pseudourostyla* sp-QDHXZ2007102801 | 94.30 | 95.62 | 93.64 | 94.68 | 96.14 | 93.03 | 94.36 | 95.88 | 92.19 | 95.87 | 92.91 | 91.74 | -- | 81.93 |
| 14 *Thigmokeronopsis stoecki* | 94.04 | 96.74 | 95.86 | 97.56 | 97.31 | 92.13 | 95.95 | 94.43 | 91.85 | 95.08 | 92.07 | 92.26 | 95.75 | -- |

NOTE.-Left low: SSrRNA gene sequences; right high: ITS1-5.8S-ITS2 region sequences.
