# Supplementary material for: Evolution of the Order Urostylida (Protozoa, Ciliophora): New Hypotheses Based on Multi-Gene Information and Identification of Localized Incongruence
Source: PLoS One. 2011 Mar 8;6(3):e17471. doi: 10.1371/journal.pone.0017471 (PMC3050893; doi:10.1371/journal.pone.0017471)
Supplement: Table S2 — Evolutionary Similarities among Dataset 9, and Expressed as Percentages. (DOC) [file pone.0017471.s003.doc]

**Table S2**

|  | 1 | 2 | 3 | 4 | 5 | 6 | 7 | 8 | 9 | 10 | 11 | 12 | 13 | 14 |
| --- | --- | --- | --- | --- | --- | --- | --- | --- | --- | --- | --- | --- | --- | --- |
| 1 *Anteholosticha eigneri* | -- | 98.05 | 97.73 | 98.03 | 97.77 | 98.89 | 98.05 | 97.13 | 97.21 | 98.05 | 98.61 | 97.76 | 98.03 | 97.76 |
| 2 *Anteholosticha gracilis* | 81.07 | -- | 99.72 | 100.00 | 99.72 | 97.76 | 100.00 | 99.15 | 98.05 | 100.00 | 99.44 | 97.49 | 100.00 | 99.72 |
| 3 *Anteholosticha manca* | 84.54 | 81.19 | -- | 99.72 | 99.44 | 97.44 | 99.72 | 99.43 | 97.73 | 99.72 | 99.15 | 97.16 | 99.72 | 99.34 |
| 4 *Apokeronopsis bergeri* | 79.70 | 83.71 | 83.32 | -- | 99.72 | 97.74 | 100.00 | 99.15 | 98.03 | 100.00 | 99.44 | 97.75 | 100.00 | 99.72 |
| 5 *Bergeriella ovata* | 84.88 | 84.95 | 87.21 | 83.71 | -- | 97.48 | 99.72 | 98.86 | 97.77 | 99.72 | 99.17 | 97.20 | 99.72 | 99.34 |
| 6 *Holosticha diademata* | 90.48 | 83.12 | 83.05 | 80.59 | 83.63 | -- | 97.76 | 96.84 | 97.50 | 97.77 | 97.76 | 97.20 | 97.75 | 97.48 |
| 7 *Metaurostylopsis* sp-QDCXM08060901 | 79.41 | 81.19 | 85.31 | 84.25 | 86.80 | 78.46 | -- | 99.15 | 98.05 | 100.00 | 99.44 | 97.49 | 100.00 | 99.72 |
| 8 *Nothoholosticha fasciola* | 81.33 | 80.00 | 84.86 | 84.27 | 87.93 | 79.80 | 84.44 | -- | 97.14 | 99.15 | 98.57 | 96.56 | 99.15 | 98.86 |
| 9 *Parabirojimia multinucleata* | 87.84 | 81.61 | 86.29 | 82.78 | 86.35 | 88.86 | 82.94 | 84.15 | -- | 98.06 | 98.05 | 95.80 | 98.04 | 97.77 |
| 10 *Pseudokeronopsis carnea* | 85.81 | 87.13 | 80.93 | 81.06 | 84.10 | 90.30 | 77.90 | 78.76 | 84.42 | -- | 99.45 | 97.21 | 100.00 | 99.72 |
| 11 *Psammomitra retractilis* | 86.87 | 82.42 | 82.62 | 83.68 | 85.26 | 87.11 | 81.39 | 82.14 | 87.37 | 85.97 | -- | 97.20 | 99.44 | 99.16 |
| 12 *Pseudoamphisiella quadrinucleata* | 88.40 | 81.68 | 80.98 | 78.74 | 82.65 | 91.96 | 77.49 | 77.59 | 86.11 | 89.66 | 85.55 | -- | 97.47 | 97.20 |
| 13 *Pseudourostyla* sp-QDHXZ2007102801 | 81.11 | 78.72 | 86.58 | 84.43 | 83.38 | 77.76 | 86.64 | 87.05 | 83.26 | 77.40 | 81.95 | 75.48 | -- | 99.72 |
| 14 *Thigmokeronopsis stoecki* | 85.55 | 83.94 | 84.05 | 83.70 | 87.26 | 86.74 | 82.21 | 83.83 | 83.59 | 86.45 | 86.32 | 84.32 | 82.56 | -- |

NOTE.-Left low: alpha-tubulin nucleotide sequences; right high: alpha-tubulin amino acid sequences.
