# Supplementary material for: Evolution of the Order Urostylida (Protozoa, Ciliophora): New Hypotheses Based on Multi-Gene Information and Identification of Localized Incongruence
Source: PLoS One. 2011 Mar 8;6(3):e17471. doi: 10.1371/journal.pone.0017471 (PMC3050893; doi:10.1371/journal.pone.0017471)
Supplement: Table S4 — Alteration of Bootstrap Support δ Depending on the Order a Particular Partition Is Added Shown for Node 2 (See Fig. 4 ). (DOC) [file pone.0017471.s005.doc]

**Table S4**

|  |  | BS |  |  |  | BS |  |  |  | BS |  |
| --- | --- | --- | --- | --- | --- | --- | --- | --- | --- | --- | --- |
| Add alpha-tubulin | Before | After | δ | Add ITS1-5.8S-ITS2 | Before | After | δ | Add SSrRNA | Before | After | δ |
| ITS1-5.8S-ITS2 | 40 | 0 | -40 | alpha-tubulin | 0 | 0 | 0 | alpha-tubulin | 0 | 0 | 0 |
| SSrRNA | 75 | 0 | -75 | SSrRNA | 75 | 49 | -26 | ITS1-5.8S-ITS2 | 40 | 49 | 9 |
| ITS1-5.8S-ITS2 + SSrRNA | 49 | 88 | 39 | alpha-tubulin + SSrRNA | 0 | 88 | 88 | alpha-tubulin + ITS1-5.8S-ITS2 | 0 | 88 | 88 |
| Mean |  |  | -25 | Mean |  |  | 21 | Mean |  |  | 32 |
