# Supplementary material for: Evolution of the Order Urostylida (Protozoa, Ciliophora): New Hypotheses Based on Multi-Gene Information and Identification of Localized Incongruence
Source: PLoS One. 2011 Mar 8;6(3):e17471. doi: 10.1371/journal.pone.0017471 (PMC3050893; doi:10.1371/journal.pone.0017471)
Supplement: Table S7 — Alteration of Bootstrap Support δ Depending on the Order a Particular Partition Is Added Shown for Node 5 (See Fig. 4 ). (DOC) [file pone.0017471.s008.doc]

**Table S7**

|  |  | BS |  |  |  | BS |  |  |  | BS |  |
| --- | --- | --- | --- | --- | --- | --- | --- | --- | --- | --- | --- |
| Add alpha-tubulin | Before | After | δ | Add ITS1-5.8S-ITS2 | Before | After | δ | Add SSrRNA | Before | After | δ |
| ITS1-5.8S-ITS2 | 91 | 68 | -23 | alpha-tubulin | 0 | 68 | 68 | alpha-tubulin | 0 | 90 | 90 |
| SSrRNA | 60 | 90 | 30 | SSrRNA | 60 | 91 | 31 | ITS1-5.8S-ITS2 | 91 | 91 | 0 |
| ITS1-5.8S-ITS2 + SSrRNA | 91 | 98 | 7 | alpha-tubulin + SSrRNA | 90 | 98 | 8 | alpha-tubulin + ITS1-5.8S-ITS2 | 68 | 98 | 30 |
| Mean |  |  | 5 | Mean |  |  | 36 | Mean |  |  | 40 |
